# The Effects of Auricular Therapy for Cancer Pain: A Systematic Review and Meta-Analysis

**DOI:** 10.1155/2020/1618767

**Published:** 2020-05-25

**Authors:** Yulan Yang, Jian Wen, Jianyun Hong

**Affiliations:** ^1^Department of Acupuncture, Maoming People's Hospital, Maoming, Guangdong, China; ^2^Department of Oncology, Maoming People's Hospital, Maoming, Guangdong, China

## Abstract

**Objective:**

This study aims to systematically assess the efficacy and safety of auricular therapy for cancer pain.

**Methods:**

A systematic search was conducted using PubMed, EMBASE, Cochrane library databases, CNKI, VIP, WanFang Data, and CBM for randomized controlled trials (RCTs). Review Manager 5.3 was used for meta-analysis.

**Results:**

Of the 275 screened studies, nine RCTs involving 783 patients with cancer pain were systematically reviewed. Compared with drug therapy, auricular therapy plus drug therapy has significant advantages both in the effective rate for pain relief (RR = 1.40; 95% CI 1.22, 1.60; *P* < 0.00001) and adverse effects rate (RR = 0.46; 95% CI 0.37, 0.58; *P* < 0.00001). And the result revealed that auricular acupuncture had superior pain-relieving effects as compared with sham auricular acupuncture (SMD = -1.45; 95% CI -2.80, -0.09; *P*=0.04). However, the analysis indicated no difference on the effective rate for pain relief between auricular therapy and drug therapy (RR = 1.24; 95% CI 0.71, 2.16; *P*=0.46).

**Conclusion:**

Our meta-analysis indicated that auricular therapy is effective and safe for the treatment of cancer pain, and auricular therapy plus drug therapy is more effective than drug therapy alone, whether in terms of pain relief or adverse reactions. However, the included RCTs had some methodological limitations; future large, rigor, and high-quality RCTs are still needed to confirm the benefits of auricular therapy on cancer pain.

## 1. Introduction

Pain is one of the most prevalent symptoms in cancer patients, when the prevalence is estimated to be more than 70% [1]. Because of its high prevalence and negative impact on patients' quality of life, pain becomes a focal point for intervention in cancer survivorship. The World Health Organization (WHO) promotes three-step analgesic method and recommends that opioids may be used as first-line treatment for moderate to severe cancer pain [2]. Although there is an increased awareness of cancer pain in the literature [3, 4], the overall effect is still not satisfactory. A meta-analyses by Deandrea et al. [5] indicated that almost a third of cancer patients failed to receive adequate treatment. In addition, there will be a series of adverse reactions after taking a lot of analgesics. Consequently, to find some more effective treatments for cancer pain, many individuals have turned their attention to other complementary and alternative medicine (CAM) [6, 7], such as acupuncture plus drug therapy, psychoeducational interventions, music interventions, Chinese herbal medicine therapy, qigong, homeopathy (Traumeel), and creative arts therapies [7].

Auricular therapy is a conventional therapy in traditional Chinese medicine, and its effect is gradually recognized. Since the 1980s, studies in auricular therapy for pain management have increased [8–12], such as for perioperative pain [11, 13, 14], dysmenorrheal [15–17], arthralgia [18], and cancer pain [19]. In order to gather and evaluate the efficacy and safety of auricular therapy for cancer pain, we conducted this systematic review and meta-analysis.

## 2. Methods

### 2.1. Search Strategy

This review was performed according to the Preferred Reporting Items for Systematic Review and Meta-Analysis (PRISMA) statement. In order to obtain relevant studies, we systematically searched PubMed, EMBASE, Cochrane library databases, the China National Knowledge Infrastructure (CNKI), Chinese Science and Technology Periodical Database (VIP), WanFang Data Information Site, and Chinese Biology Medicine Disc (CBMdisc) from inception to February 21, 2020. The following Search terms were used for cancer pain: “Cancer” OR “Tumor” OR “carcinoma” OR “Oncological” OR “Malignancy”AND “Ache” OR “Aches” OR “Physical Suffering” OR “Suffering, Physical”. While the following Search terms were used for auricular therapy: “acupuncture, Auricular” OR “auricular therapy” OR “auricular needle” OR “auricular acupressure” OR “ear acupuncture” OR “ear acupressure” OR “acupuncture ear” OR “otopoint” OR “otoneedle” OR “auriculoacupuncture” OR “auriculotherapy”. The specific search strategy is shown in Table 1.

### 2.2. Study Selection

#### 2.2.1. Inclusion Criteria of Studies

Inclusion criteria were (1) randomized controlled trials (RCTs) in English or Chinese, (2) adult patients diagnosed with any stage of cancer who experienced cancer pain, and (3) the intervention of auricular therapy alone or plus drug therapy. The auricular therapy refers to auricular needle, auricular acupressure, auricular point injection, auricular acupuncture, and auricular point sticking with seed or pellet attachments, while the control group received treatment with drug therapy, or placebo treatment.

#### 2.2.2. Exclusion Criteria of Studies

Exclusion criteria were (1) no RCTs, (2) case reports, review articles, and animal experiments, and (3) trials that studied pain which cannot be clearly attributed to cancer, for example, trials that involve patients after surgical resection of tumors or other reasons.

Two authors (Yang and Wen) independently screened literatures from databases in this review. Any inconsistency was discussed and resolved with the third author (Hong).

Of the 275 screened literatures, 84 duplicates were removed. And then, case reports, animal experiments, and review articles were excluded by reading titles and abstracts. Finally, inconsistent literatures of intervention and outcome measures were excluded by reading the full texts.

**Figure 1:**
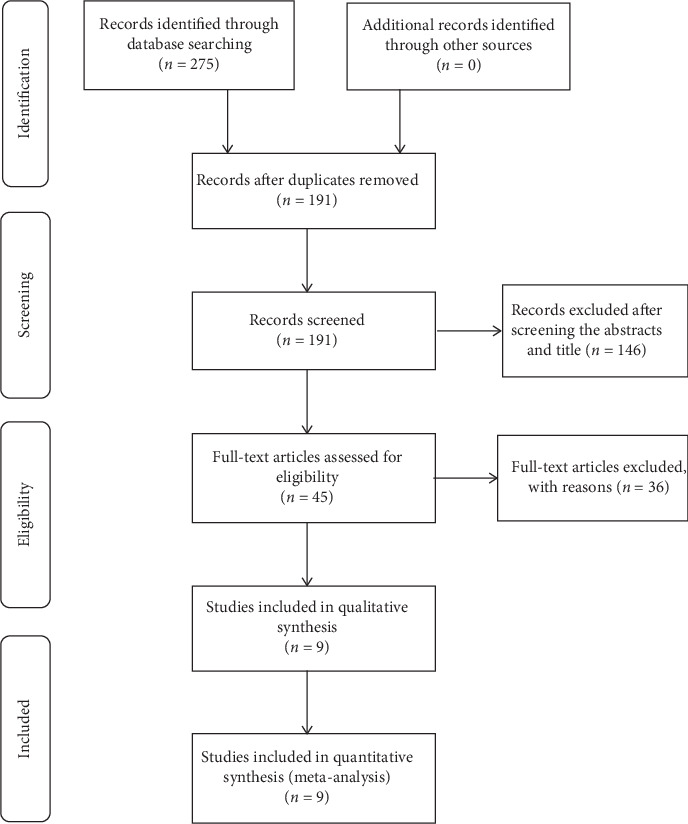
Flow chart for the publication selection process.

The PRISMA flow chart showed the study selection process in Figure 1.

### 2.3. Data Extraction

Two authors (Yang and Wen) independently reviewed the studies included. The following data was extracted from the trials using predesigned form: first author name, publication year, sample sizes, characteristics of patients, randomized method, interventions, outcome measures, and adverse events. If the information was incomplete, we tried to contact the author to acquire it.

### 2.4. Risk of Bias Assessment

The quality of RCTs included was appraised based on guidance in the Cochrane Handbook for Systematic Reviews of Interventions [20]. The included studies were independently evaluated by two reviewers in terms of seven aspects: random sequence generation, allocation concealment, blinding of participants and personnel, blinding of outcome assessment, incomplete outcome data, selective reporting, and other sources of bias. Each study was scored as low, unclear, or high risk of bias.

### 2.5. Data Analysis

Statistical analysis was performed with Review Manager 5.3 of Cochrane Library. *P* < 0.05 was considered statistically significant. Dichotomous data was analyzed using risk ratio (RR) and 95% confidence intervals (CIs). Continuous data was analyzed using a mean difference (MD) and standard mean difference (SMD) with 95% CI. We evaluate heterogeneity depending on both a chi-squared test (Cochrane's *Q* statistic) and an I^2^ statistic. If there was substantial heterogeneity among studies, a random-effects model was used. Conversely, a fixed-effects model was used. Continuous data reported using mean and range values was calculated or estimated performing calculations described by Hozo et al. [21] and Luo et al. [22].

## 3. Results

### 3.1. Characteristics of Studies Included in the Review

Of the 275 screened studies, nine RCTs involving 783 patients with cancer pain were systematically reviewed. Two [23, 24] were published in English, and seven [25–31] were published in Chinese. In the treatment groups, four studies [23, 24, 27, 28] adopted auricular therapy alone; two [23, 24] used auricular acupuncture and two [27, 28] used auricular point sticking, and ear acupoint injection, respectively. And the other five studies [25, 26, 29–31] utilized auricular therapy (including ear acupoint injection, auricular press needle, ear point embedding, and auricular point sticking) plus drug therapy, while, in the control groups, two studies [23, 24] treated participants with sham auricular acupuncture, and the others adopted drug therapy. The characteristics of these studies are presented in Table 2.

### 3.2. Quality Assessment

**Figure 2:**
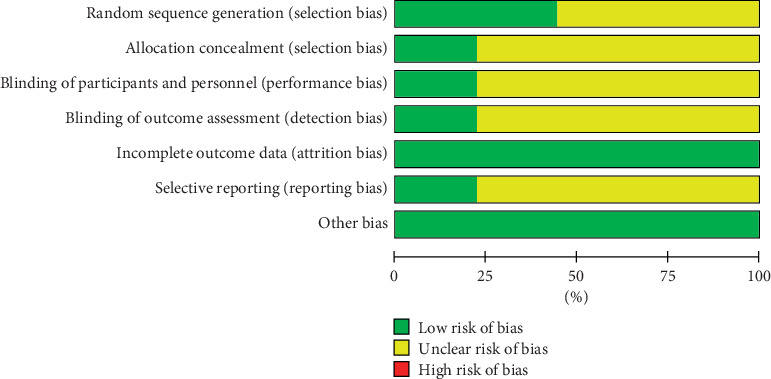
Risk of bias graph: review authors' judgments about each risk of bias item presented as percentages across all included studies.

**Figure 3:**
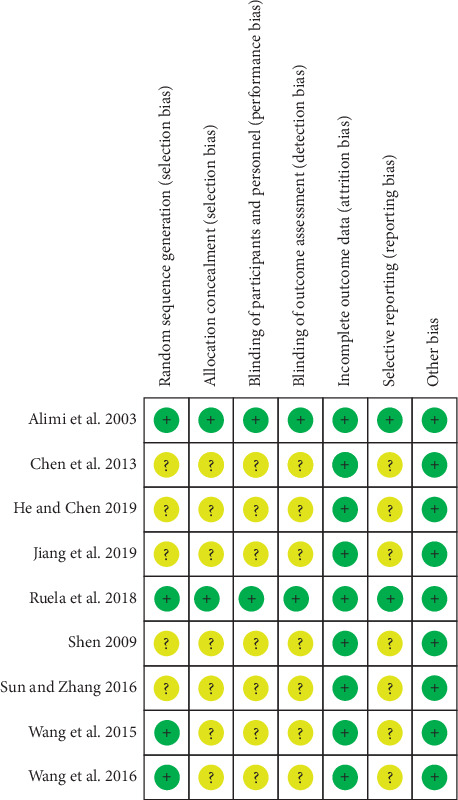
Risk of bias summary: review authors' judgments about each risk of bias item for each included study.

Four included RCTs [23, 24, 30, 31] were rated as low risk of bias, which reported adequate methods of random sequence generation, while the others were considered to have an unclear risk of bias due to the lack of any description. Two RCTs [23, 24] that reported allocation concealment were rated as low risk of bias. Seven RCTs [25–31] did not have any described method and so were assessed as unclear risk of allocation concealment. Among the nine RCTs, two were double-blinded [23, 24], while others were not blinded [25–31]. All of the nine RCTs reported all expected outcomes and hence at low risk of bias for selective reporting. Overall, two studies [23, 24] were identified as high quality.  Figure 2 and 3 show summaries of the risk of bias.

### 3.3. Result Analysis

#### 3.3.1. Primary Outcome

**Figure 4:**
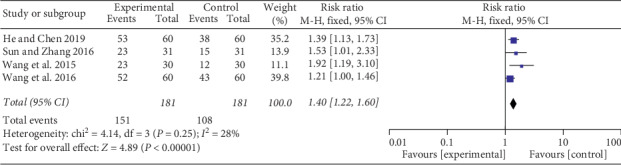
Forest plot of auricular therapy plus drug therapy on the effective rate for pain relief compared with drug therapy alone.

**Figure 5:**
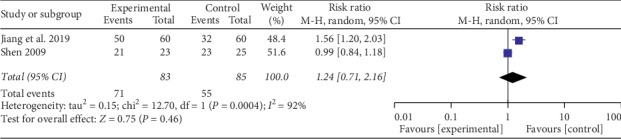
Forest plot of auricular therapy on effective rate for pain relief compared with drug therapy.

*(1) Effective Rate for Pain Relief*. Six studies [26–31] provided data on the effective rate for pain relief. Four studies [26, 29–31] compared the effects of auricular therapy plus drug therapy with drug therapy alone. Since there was no significant heterogeneity (Chi^2^ = 4.14, *P*=0.25, I^2^ = 28%, Figure 4), the statistical analysis was performed using a fixed-effects model. The results detected significant effects of auricular therapy plus drug therapy in reducing cancer pain (RR = 1.40; 95% CI 1.22, 1.60; *P* < 0.00001). However, a subgroup analysis focused on the other two studies [27, 28] indicated no difference on the effective rate for pain relief between auricular therapy and drug therapy (RR = 1.24; 95% CI 0.71, 2.16; *P*=0.46; Figure 5). The data analysis was represented by a random-effects model due to heterogeneity (Chi^2^ = 12.70, *P*=0.0004; I^2^ = 92%).

#### 3.3.2. Secondary Outcome

**Figure 6:**
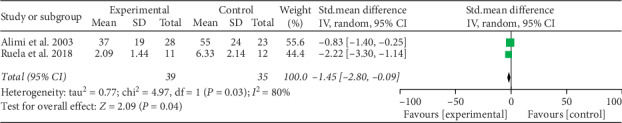
Forest plot of auricular therapy for the treatment of cancer pain compared with sham auricular therapy.

*(1) Pain Score*. Alimi et al. [23] and Ruela et al. [24] treated participants by setting up auricular acupuncture group and sham auricular acupuncture group; the pain scores were carried out after treatment. The results indicated that auricular acupuncture could reduce the pain score of cancer patients. And there was a statistical difference (SMD = −1.45; 95% CI −2.80, −0.09; *P*=0.04; Figure 6) between the two groups. We used a random-effects model due to heterogeneity (Chi^2^ = 4.97, *P*=0.03; I^2^ = 80%).

**Figure 7:**
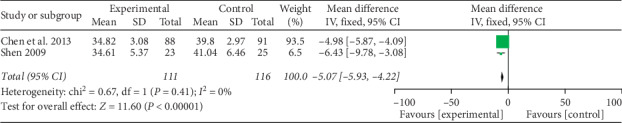
Quality of life: experimental groups compared with control groups.

*(2) Quality of Life*. Among the nine studies, two provided data related to the quality of life [25, 28]. Meta-analysis of the two RCTs demonstrated a significant difference (MD = −5.07; 95% CI −5.93, −4.22; *P* < 0.00001; Figure 7) between experimental groups compared with control groups on improving the patients' quality of life, with no heterogeneity (Chi^2^ = 0.67, *P*=0.41; I^2^ = 0%).

**Figure 8:**
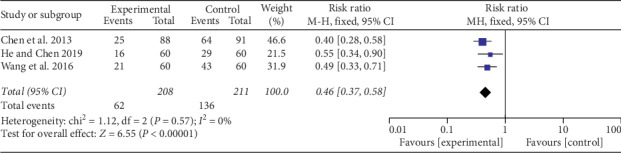
Adverse effects rate: auricular therapy plus drug therapy versus drug therapy alone.

*(3) Adverse Effects Rate*. Of the nine included RCTs, six trials [24–26, 28, 30, 31] assessed adverse effects, while others [23, 27, 29] did not mention any. Ruela et al. [24] reported that there were no adverse reactions in auricular acupuncture. And the other two studies by Shen [28] and Wang et al. [30] were excluded from the analysis due to lack of data. Analysis results of the remaining three studies [25, 26, 31] showed significant advantages of auricular therapy plus drug therapy in reducing adverse effects (RR = 0.46; 95% CI 0.37, 0.58; *P* < 0.00001; Figure 8). A fixed-effects model was used for statistical analysis because of no significant heterogeneity (Chi^2^ = 1.12, *P*=0.57; I^2^ = 0%).

## 4. Discussion

The choice of analgesic depends on the severity of the pain; as the pain increases, so does the strength of the recommended analgesic [32]. Continuous administration of the drug itself leads to an attenuation of effect [33]. Therefore, there is an urgent need for new pain-relieving strategies in patients with insufficient pain relief of cancer pain.

This study aims to systematically assess the efficacy and safety of auricular therapy for cancer pain. Our systematic review and meta-analysis of nine RCTs involving 783 patients compared the efficacy and safety of auricular therapy and drug therapy or sham auricular therapy for cancer pain.

In this review, three subgroups compared the efficacy of reducing the intensity of cancer pain. First was auricular therapy plus drug therapy versus drug therapy. The results showed auricular therapy plus drug therapy was more effective in relieving pain intensity (RR = 1.40; 95% CI 1.22, 1.60; *P* < 0.00001, Figure 4). Second was auricular acupuncture versus sham auricular acupuncture. Alimi et al. [23] and Ruela et al. [24] demonstrated in their studies that auricular acupuncture was an effective method for mitigating pain. And there was a statistical difference on cancer pain reduction (SMD = −1.45; 95% CI −2.80, −0.09; *P*=0.04; Figure 6). Third was auricular therapy compared with drug therapy. Nevertheless, the result failed to display a significant difference (RR = 1.24; 95% CI 0.71, 2.16; *P*=0.46; Figure 5). All of the studies included in this review reported pain intensity measured by numerical rating scale (NRS) or visual analogue scale (VAS), both on a scale from 0 to 10, with 0 indicating no pain and 10 reflecting the most serious pain imaginable [34]. The subjectivity and multidimensional nature of the pain experience make the pain assessment have individual differences. In addition, we did not classify the pain of different cancers, which may have biased the results. Therefore, further rigorous trials are needed to confirm our results.

In our review, data of quality of life in posttreatment were pooled from two RCTs. Meta-analysis of the two RCTs [25, 28] demonstrated a significant difference (MD = −5.07; 95% CI −5.93, −4.22; *P* < 0.00001; Figure 7) between experimental groups compared with control groups. However, only 2 trials were included in the analysis; thus, larger RCTs are needed to verify these findings.

Two experiments [25, 28] reported that auricular therapy can reduce the onset time and increase the maintenance time of analgesia. Three trials [24, 25, 28] mentioned that auricular therapy helps reduce the consumption of analgesics. Due to the lack of data, we did not carry out the comparative analysis.

Our meta-analysis confirmed that auricular therapy was safe, effective, and inexpensive for cancer pain. Not only may auricular therapy have an effect on cancer pain relief, but also it may have other benefits including the potential reduction of analgesics and the benefits of improving the quality of life as well as treatment compliance. The combined use of auricular therapy and drug therapy is significantly superior compared to that of drug therapy. The results will provide some clues for the use of auricular therapy in cancer pain. We propose auricular therapy for cancer pain, when the adverse effects of analgesic are serious or the recommended strength of analgesic cannot effectively relieve the pain. In the future research, we should pay more attention to the standardization of auricular therapy, the selection of acupoints and the treatment course, and follow-up to the long-term effect.

It must be acknowledged that our meta-analysis has several limitations. Firstly, the diversity of auricular therapy might increase the risk of bias. Because the auricular therapy was not consistent across studies, we could not estimate the correlation between the difference of auricular therapy and its therapeutic effectiveness. Secondly, it may cause certain degree of publication bias, because only two of the included studies were published in English and seven were published in Chinese. Thirdly, the sample size of the RCT conducted by Ruela et al. [24] is small, eleven in the experimental group and twelve in the control group, which may lead to statistical bias. Fourthly, the lack of description of the random sequence, blinding, and allocation concealment might have biased the results. Lastly, no studies reported information of follow-up, and therefore, the long-term efficacy remains to be confirmed.

## 5. Conclusion

Our meta-analysis indicated that auricular therapy is effective and safe for the treatment of cancer pain, and auricular therapy plus drug therapy is more effective than the drug therapy alone, whether in terms of pain relief or adverse reactions. However, the included RCTs had some methodological limitations, large, rigor, and high-quality. RCTs are still needed to confirm the benefits of auricular therapy on cancer pain.

## Figures and Tables

**Table 1:** Search strategy in EMBASE up till February 21, 2020 (similar search run in other databases).

1.	“neoplasm”/exp
2.	“cancer”:ti, ab, kw OR “tumor”: ti, ab, kw OR “carcinoma”: ti, ab, kw OR “oncological”: ti, ab, kw OR “malignancy”: ti, ab, kw
3.	#1 OR #2
4.	“pain”/exp
5.	“ache”: ti, ab, kw OR “aches”: ti, ab, kw OR “physical suffering”: ti, ab, kw OR “suffering, physical”: ti, ab, kw
6.	#4 OR #5
7.	“Auricular acupuncture”/exp
8.	“Acupuncture, auricular”: ti, ab, kw OR “auricular therapy”: ti, ab, kw OR “auricular needle”: ti, ab, kw OR “auricular acupressure”: ti, ab, kw OR “ear acupuncture”: ti, ab, kw OR “ear acupressure”: ti, ab, kw OR “acupuncture ear”: ti, ab, kw OR “otopoint”: ti, ab, kw OR “otoneedle”: ti, ab, kw OR “auriculoacupuncture”: ti, ab, kw OR “auriculotherapy”: ti, ab, kw
9.	#7 OR #8
10.	“Randomized controlled trial”: ti, ab, kw OR “randomized”: ti, ab, kw OR “placebo”: ti, ab, kw
11.	#3 AND #6 AND #9 AND #10

**Table 2:** Characteristics of the included studies.

Author (year)	Diagnosis	Cancer pain type	Sample sizes (M/F)	Age (year)	Randomized method	Interventions	Acupoint selection	Course of treatment	Outcome measures
Alimi et al. 2003 [23]	Head and neck cancer, breast cancer, Lung cancer, and others	Attained a pain level evaluated at 30 mm or more on a VAS graduated from 0 to 100 mm	E: 28	Not reported	Computer software	E : Auricular acupuncture (at points where an electrical response had been detected)	At points where an electrodermal signal had been detected	Two courses	Pain relief measured by VAS
			C: 23			C : Placebo auricular acupuncture (at placebo points)			
Ruela et al. 2018 [24]	Breast cancer and others	Pain ≥ four in the numerical pain scale	E: 11	E: 58.27 ± 10.09	Simple	E : Auricular acupuncture (5–7 needles, 40 min/time, 7 d/session)	E: Shenmen, kidney, sympathetic, muscle relaxation and the energy balance points	Eight sessions	Pain relief by NRS
			C: 12	C: 52.08 ± 7.99	Randomization (biased coin method)	C : Auricular acupuncture at fixed placebo points (2 needles, 40 min/time, 7 d/session)	C: The eye and trachea points		
Chen et al. 2013 [25]	Stage IV tumor	Moderate to severe cancer pain	E: 88 (46/42)	E: 58.32 ± 10.54	Not reported	E : Ear acupoint injection (IS) (2–3 points, 1/day, 7 days), plus drug	Corresponding points of main organs invaded by cancer	One course	Pain relief measured by NRS/Onset and maintenance time of analgesia/quality of life/adverse effects rate
			C: 91 (47/44)	C: 60.13 ± 9.76		C : Drug (three-step analgesic ladder)			
He and Chen 2019 [26]	Lung cancer, stomach cancer, liver cancer, esophageal cancer	Moderate to severe cancer pain	E: 60 (35/25)	E: 58.59 ± 3.16	Not reported	E : Auricular point sticking (7points, 2 min/point, 5/d, change/5 d), plus drug	Rectum, large intestine, subcortical, sanjiao, spleen, shenmen, sympathetic	Not reported	Pain relief measured by NRS/adverse effects rate
			C: 60 (39/21)	C: 59.58 ± 3.55		C : Drug (morphine, 10 mg/time, 2/d)			
Jiang et al. 2019 [27]	Malignant tumor or bone metastasis	Moderate to severe cancer pain	E: 60 (29/31)	E: 45.67 ± 22.24	Not reported	E : Auricular point sticking (5–6 points, 3-5 min/point, total 3 weeks)	Subcortical, shenmen, liver, sanjiao, sympathetic	Three weeks	Pain relief measured by NRS/quality of life/treatment compaliance
			C: 60 (31/29)	C: 44.92 ± 24.13		C : Drug (Oxycontin, 10 mg/time, 2/d, total 3 weeks)			
Shen 2009 [28]	Advance d malignant tumor	Moderate to severe cancer pain	E: 23 (12/11)	E: 59.43 ± 10.43	Not reported	E : Ear acupoint-injection (IS) (2–3 points, 1/day, 7 days.)	Corresponding points of main organs invaded by cancer	One course	Pain relief measured by NRS/onset and maintenance time of analgesia/quality of life
			C: 25 (11/14)	C: 60.24 ± 9.92		C : Drug (Oxycontin, 5–10 mg/time, 2/d.)			
Sun and Zhang 2016 [29]	Lung cancer, liver cancer, breast cancer, esophageal cancer, pancreatic cancer, gastric cancer, colorectal cancer	Moderate cancer pain	E: 31 (17/14)	E: 68.40 ± 4.72	Not reported	E : Auricular press needle (3–5 points, change/5 d, 10 days), plus drug.	Subcortical, shenmen, sympathetic, upper ear root, lower ear root	One course	Pain relief measured by NRS
			C: 31 (15/16)	C: 65.90 ± 5.06		C : Drug (tramadol hydrochloride, 100 mg/time, Q12H, 10 days)			
Wang et al. 2015 [30]	Malignant tumor or bone metastasis	Moderate to severe cancer pain	E: 30 (23/27)	2.87 ± 10.96	Random number table	E : Ear point embedding (several points, 2–3 min/point, 3/d), plus drug.	Stomach, liver, spleen, cardia, sympathetic, shenmen, subcortical + corresponding points of main organs invaded by cancer	Not reported	Pain relief measured by NRS/adverse effects rate
			C: 30 (23/27)			C : Drug			
Wang et al. 2016 [31]	Advanced malignant tumor	Moderate to severe cancer pain	E: 60 (39/21)	E: 61.7 ± 7.1	Random number table	E : Auricular point sticking (7 points, 2 min/point, 3–4/d, 7 days), plus drug.	Shenmen, sympathetic, subcortical, spleen, sanjiao, rectum, large intestine	One course	Pain relief measured by NRS/adverse effects rate
			C: 60 (37/23)	C: 62.1 ± 6.7		C : Drug (7 days)			

M, male; F, female; E, experimental group; C, control group; NRS, numerical rating scale; VAS, visual analogue scale.
